# Purification and Biochemical Characterization of Three Myotoxins from *Bothrops mattogrossensis* Snake Venom with Toxicity against *Leishmania* and Tumor Cells

**DOI:** 10.1155/2014/195356

**Published:** 2014-03-03

**Authors:** Andréa A. de Moura, Anderson M. Kayano, George A. Oliveira, Sulamita S. Setúbal, João G. Ribeiro, Neuza B. Barros, Roberto Nicolete, Laura A. Moura, Andre L. Fuly, Auro Nomizo, Saulo L. da Silva, Carla F. C. Fernandes, Juliana P. Zuliani, Rodrigo G. Stábeli, Andreimar M. Soares, Leonardo A. Calderon

**Affiliations:** ^1^Centro de Estudos de Biomoléculas Aplicadas à Saude, CEBio, Fundação Oswaldo Cruz, Fiocruz Rondônia e Departamento de Medicina, Núcleo de Saúde, Universidade Federal de Rondônia, UNIR, 76812–245 Porto Velho, RO, Brazil; ^2^Fundação Oswaldo Cruz, Fiocruz Rondônia, 76812–245 Porto Velho, RO, Brazil; ^3^Departamento de Biologia Celular e Molecular, Instituto de Biologia, Universidade Federal Fluminense, UFF, 24020-141 Niterói, RJ, Brazil; ^4^Faculdade de Ciências Farmacêuticas, FCFRP, Universidade de São Paulo, USP, 14040-903 Ribeirão Preto, SP, Brazil; ^5^Universidade Federal de São João del Rei, UFSJ, 36420-000 Ouro Branco, MG, Brazil

## Abstract

*Bothrops mattogrossensis* snake is widely distributed throughout eastern South America and is responsible for snakebites in this region. This paper reports the purification and biochemical characterization of three new phospholipases A_2_ (PLA_2_s), one of which is presumably an enzymatically active Asp49 and two are very likely enzymatically inactive Lys49 PLA_2_ homologues. The purification was obtained after two chromatographic steps on ion exchange and reverse phase column. The 2D SDS-PAGE analysis revealed that the proteins have pI values around 10, are each made of a single chain, and have molecular masses near 13 kDa, which was confirmed by MALDI-TOF mass spectrometry. The N-terminal similarity analysis of the sequences showed that the proteins are highly homologous with other Lys49 and Asp49 PLA_2_s from *Bothrops* species. The PLA_2_s isolated were named BmatTX-I (Lys49 PLA_2_-like), BmatTX-II (Lys49 PLA_2_-like), and BmatTX-III (Asp49 PLA_2_). The PLA_2_s induced cytokine release from mouse neutrophils and showed cytotoxicity towards JURKAT (leukemia T) and SK-BR-3 (breast adenocarcinoma) cell lines and promastigote forms of *Leishmania amazonensis*. The structural and functional elucidation of snake venoms components may contribute to a better understanding of the mechanism of action of these proteins during envenomation and their potential pharmacological and therapeutic applications.

## 1. Introduction

Snake venoms contain a complex mixture of components with a wide range of biological and pharmacological activities. More than 90% of their dry weight is composed of proteins, including a variety of enzymes, such as phospholipases A_2_, proteases (metallo and serine), L-amino acid oxidases, esterases, as well as many other nonenzymatic proteins and peptides [1–3]. These proteins and peptides can be grouped into a small number of superfamilies based on remarkable similarities in their primary, secondary, and tertiary structures, while showing distinct pharmacologic effects [3].

One important protein superfamily present in all snake venoms is phospholipase A_2_ (PLA_2_, E.C. 3.1.1.4). PLA_2_s are a class of heat-stable and highly homologous enzymes, which catalyze the hydrolysis of the 2-acyl bond of cell membrane phospholipids releasing free fatty acids such as arachidonic acid and lysophospholipids. PLA_2_s have been characterized as the major component of snake venoms, being responsible for several pathophysiological effects caused by snake envenomation, such as neurotoxic, cardiotoxic, myotoxic, cytotoxic, hypotensive, and anticoagulant activities triggering an intense inflammatory reaction with the release of cytotoxins and eicosanoids [4–6]. PLA_2_'s involvement in a variety of inflammatory diseases and accidents caused by venomous animals has raised medical and scientific interest in this enzyme [7, 8].

Myotoxic PLA_2_s from the *Bothrops* species are composed of approximately 110 to 135 amino acid residues and can be divided into two groups: “classical”, which contain an aspartate residue at position 49 (Asp49) and catalyze ester bond hydrolysis at the glycerophospholipid *sn*-2 position in a Ca^2+^-dependent manner; and “variant”, which contain a lysine residue at the same position (Lys49). This substitution affects the ability of these proteins to bind to Ca^2+^, which is an essential cofactor for catalysis, leading to decreased or no catalytic activity [1–5, 7, 9, 10].

The *Bothrops mattogrossensis* snake belongs to the *Bothrops neuwiedi* complex [11]. This snake is found in the eastern region of South America including Bolivia, Brazil, Southeast Peru, Paraguay, Uruguay, and Argentina [12]. The present study describes for the first time the isolation, identification, and functional characterization of three myotoxic phospholipases A_2_, named: BmatTX-I (Lys49 PLA_2_-like), BmatTX-II (Lys49 PLA_2_-like), and BmatTX-III (Asp49 PLA_2_) and evaluates their activity against *Leishmania* and tumor cells.

## 2. Materials and Methods

### 2.1. Venom

The venom from the *Bothrops mattogrossensis* snake was acquired from Serpentário de Proteínas Bioativas, Batatais-SP, Brazil. This study was authorized by Instituto Brasileiro do Meio Ambiente e dos Recursos Naturais Renováveis—IBAMA, Instituto Chico Mendes de Conservação da Biodiversidade—ICMBio (number: 27131-1) and Conselho de Gestão do Patrimônio Genético—CGEN/Brazil (number 010627/2011-1).

### 2.2. Animals

Swiss male mice (18–20 g) were used. These animals were housed in temperature-controlled rooms and received water and food *ad libitum* until used. Animal care was in accordance with the guidelines of the Brazilian College for Animal Experimentation (COBEA) and was approved by the Committee of Ethics on Animal Utilization in Research from the Institute of Research of Tropical Pathologies (IPEPATRO/FIOCRUZ-Rondônia) (protocol number 2012/1).

### 2.3. *Bothrops mattogrossensis* Venom Fractioning

Around 250 mg of *B. mattogrossensis* venom was eluted in 1.2 mL of sterile deionized water and centrifuged at 1,530 ×g for 10 minutes and then submitted to cation exchange chromatography in an Akta purifier 10 (GE-Healthcare) using a CM-Sepharose column (27 × 300 mm, GE Healthcare), equilibrated with 50 mM ammonium bicarbonate buffer, pH 8.0, and eluted with a linear gradient of 0–100% 500 mM ammonium bicarbonate, pH 8.0 at a flow rate of 5.0 mL/minute. The fractions were monitored at an absorbance of 280 nm, collected manually, identified, lyophilized, and stored at −20°C. All fractions were submitted to SDS-PAGE and enzymatic activity analyses. The isolation of the three PLA_2_s was obtained by liquid chromatography using a Discovery C18 column (25 × 4.6 mm, Supelco) equilibrated with deionized water with 0.1% trifluoroacetic acid (v/v) and eluted with ten volumes of a linear gradient of 0–100% acetonitrile with 0.1% trifluoroacetic acid (v/v) at a flow rate of 1.0 mL/minute. The elution was monitored at an absorbance of 280 nm, manually collected, lyophilized, and stored at −20°C. In order to determine protein concentration, the Bradford method (Bio-Rad) was used with bovine serum albumin (BSA) as a standard [14].

### 2.4. Monodimensional Electrophoresis (SDS-PAGE)

Polyacrylamide gel electrophoresis (PAGE) in the presence of sodium dodecyl sulfate (SDS) was conducted using the method described by Laemmli [15]. The electrophoresis was carried out at 15 mA and 5 W using a conventional molecular weight standard (BioLabs) and a Lys49-PLA_2_ homologue isolated from *B. jararacussu* snake venom (BthTX-I) as markers. The gel was stained with Coomassie Brilliant Blue (CBB) G-250 and images of the gel were obtained using *Image Scanner *(GE Healthcare).

### 2.5. Bidimensional Electrophoresis (2D-SDS-PAGE) and pI Determination

The protein fractions were submitted to electrofocusing in 13 cm strips with pHs ranging from 3 to 10 in a nonlinear form, using an Ettan IPGphor 3 IEF System (GE Healthcare). At the end of the electrofocusing, the strips containing the PLA_2_s were equilibrated and transferred to a 12.5% polyacrylamide gel where they were separated according to molecular mass. The electrophoretic run was performed at 25 mA and 100 W during 5.5 hours and at the end of the protein separation the gel was stained with Coomassie Brilliant Blue (CBB) G-250 and images of the gel were captured using *Image Scanner *(Amershan Bioscience).

### 2.6. Mass Spectrometry (MALDI-TOF)

The samples (1 *μ*L) were mixed with a matrix solution composed of sinapinic acid and acetonitrile with 0.1% trifluoroacetic solubilized at a 1 : 1 ratio. The average mass of the protein was obtained in a MALDI-TOF system (Shimatzu Biotech), operated in linear mode using insulin (5,734.5 Da), cytochrome C (12,361.9 Da), apomyoglobin (16,952.2 Da), aldolase (39,212.2 Da), and albumin (66,430.0 Da) as calibrators. The mass spectra obtained were submitted to automatic baseline subtraction.

### 2.7. N-Terminal Sequencing and Similarity Search

The amino terminal sequence of each isolated PLA_2_ previously determined by automated Edman degradation was used to search for sequence similarity. Approximately 50 *μ*g of each isolated PLA_2_ corresponding to approximately 2 nmols/mL sample was submitted for N-terminal amino acid sequencing using the automated Edman degradation method [13] on a PPSQ-33A (Shimadzu) automatic sequencer. A sequence similarity search and multiple sequence alignment were performed in the SWISS-PROT/TREMBL database using the programs FASTA, BLAST, and CLUSTALW2.

### 2.8. Functional Characterization

#### 2.8.1. Phospholipase Activity with 4-Nitro-3(octanoyloxy)benzoic (4N3OBA)

Phospholipase activity of the three PLA_2_s was assayed following the protocols described by Holzer and Mackessy [16], modified for 96-well plates. The standard assay mixture contained 200 *μ*L of buffer (10 mM Tris-HCl plus 10 mM CaCl_2_, and 100 mM NaCl, pH 8.0), 20 *μ*L of 4-nitro-3(octanoyloxy)benzoic (4N3OBA – Biomol, EUA), 20 *μ*L of deionized water, and 20 *μ*L of PLA_2_ (5 *μ*g). After the addition of the PLA_2_s, the mixture was incubated for 30 minutes at 37°C and the absorbance was determined at 425 nm using an Eon (Biotek) microplate spectrophotometer, for 3-minute intervals. The enzymatic activity was expressed as the reaction's initial velocity (*V*_*o*_) calculated based on the increase in absorbance.

#### 2.8.2. Phospholipase Activity with Fluorescent Substrate

Phospholipase A_2_'s (PLA_2_) enzymatic activity was evaluated through the hydrolysis of synthetic fluorescent phospholipid, using the fluorescent substrate Acyl 6 : 0 NBD phospholipid, NBD-phosphatidylcholine (NBD-PC) (Avanti Polar Lipids Inc., Alabaster, AL, USA). The assay was performed in a spectrofluorometer (Shimadzu, RF-5301PC, software RFPC) with excitation and emission wavelengths of 460 and 534, respectively. The enzymatic activity of each *B. mattogrossensis* chromatographic fraction (7, 12, 15, 16, 17, and 20) was evaluated over 250 seconds, after the addition of substrate (3.3 *μ*g/mL, final concentration) in a reaction media containing 50 mM Tris-HCl and 8 mM CaCl_2_ at pH 7.5 at room temperature.

#### 2.8.3. Platelet Aggregation

A platelet aggregation assay was carried out according to the process described by Fuly et al. [17], with modifications. Platelet aggregation was monitored in an Aggregometer (Chrono Log model 490 2D, Havertown, USA) using Platelet-Rich-Plasma (PRP). PRP was obtained from human whole blood of health volunteers (CAAE: 14204413.5.0000.0011). BmatTX-III (rechromatography fraction 15) was incubated with PRP for five minutes at 37°C being stirred constantly, and then, platelet aggregation was triggered by the addition of ADP (15 *μ*M) or collagen (16 *μ*g/mL). Assays were performed at 37°C in siliconized glass cuvettes in a final volume of 300 µL. Control experiments were performed by adding agonists in the absence of peak 15. One hundred percent (100%) platelet aggregation was obtained with a supramaximal concentration of each agonist and determined 6 minutes after the addition of each, while PRP's light transmittance showed 0% aggregation.

#### 2.8.4. Hemorrhage Activity

Mice were injected intradermally in the dorsal region with 50 *μ*g of crude venom dissolved in 50 *μ*L of physiological solution [18]. Controls received 50 *μ*L of physiological solution in identical conditions. After 3 hours, the animals were euthanized through cervical dislocation and the skin was removed. The hemorrhagicactivitywas expressed as size of thehemorrhagic area on the inner surface measured in mm^2^.

#### 2.8.5. Coagulation Activity and Minimal Coagulation Dose (MCD) Determination

Coagulation activity was tested using a methodology previously described by Gené et al. [19]. In brief, 200 *μ*L of plasma from mice was distributed in a 96-well plate, and 10 *μ*L of crude venom containing different concentrations (0.312, 0.625, 1.25, 2.5, 5, and 10 *μ*g) of proteins was added. The plate was placed in a thermostatically controlled environment and the optical density was measured every 3 seconds at 600 nm using an Eon microplate spectrophotometer (Biotek) in order to evaluate the smallest concentration of venom able to coagulate 200 *μ*L of plasma/minute.

#### 2.8.6. Proteolytic Activity

Proteolytic activitywas ascertained using azocasein (Sigma) as a substrate according to the procedure described by Charney and Tomarelli [20]. Azocasein solubilized in distilled water (150 *μ*L) was added to 7 *μ*L of the crude venom in different protein concentrations (0.312, 0.625, 1.25, 2.5, 5, and 10 *μ*g) and then the mixture was incubated in a water bath at 37°C for 1 hour. The reaction stopped when 150 *μ*L of 20% (m/v) trichloroacetic acid was added, which was followed by incubation for 30 min and centrifugation at 10,000 ×g for 10 more minutes. Then, 100 *μ*L of the supernatant was transferred to a multiple-well plate and the absorbance was measured at 440 nm using an Eon microplate spectrometer (Biotek) and one enzymatic unit (U) was defined as the amount of enzyme necessary to increase the absorbance by 0.05.

#### 2.8.7. Myotoxic Activity

Myotoxic activity was determined by measuring the creatine kinase (CK) activity in the plasma [21]. Groups of mice were injected in the gastrocnemius muscle with 25 *μ*g of isolated myotoxins diluted in 50 *μ*L of phosphate buffered saline (PBS). Negative controls received an injection of 50 *μ*L of PBS. Three hours after the injections, aliquots of mice blood were collected from the caudal vein, in heparinized capillaries and centrifuged at 1,530 ×g for 20 minutes. Creatine kinase's enzymatic activity was determined using the CK-NAC kinetic kit (Bioclin, Brazil) according to the manufacturer's protocol. Absorbance was measured for 3 minutes at 37°C, in a spectrophotometer at 340 nm. Enzymatic activity was expressed in units/liter (U/L) and each unit consists of the result of the phosphorylation of one nanomol of creatine per minute.

#### 2.8.8. Neutrophil Viability

Neutrophils were collected 6 hours after the intraperitoneal (IP) injection of 1.5 mL of 3% thioglycollate sterile solution according to the method previously described by Call et al. [22]. The animals were euthanized in order to collect the cells and the peritoneal cavity was washed with 3 mL of PBS. The predominance of neutrophils in the liquid obtained was confirmed by microscopic analysis with glass slides stained with a panoptic dye. The peritoneal neutrophils obtained were suspended in an RPMI culture medium (Gibco-BRL) supplemented with gentamicin (100 *μ*g/mL), L-glutamine (2 mM), and 10% bovine fetal serum (SFB) in order to obtain 2 × 10^5^ cells/100 *μ*L. Next, cellular viability was assayed over 1, 12, and 24 h at 37°C, and 5% CO_2_ in which the cells were incubated in a 96-well plate with the previously isolated PLA_2_s at a concentration of 12 *μ*g/mL using RPMI as a negative control. After this, the samples were centrifuged and the supernatant removed. Cellular viability was determined using the MTT method [23].

#### 2.8.9. Quantification of Cytokine

EIA was used to evaluate IL-1*β* cytokine as described by Schumacher et al. [24]. Briefly, the neutrophils (2 × 10^5^ cells/200 *μ*L) were either incubated with the isolated proteins at 3, 6, and 12 *μ*g/mL concentrations (experimental group) or with PMA (positive control group) or with RPMI (negative control) and kept for 12 and 24 hours at 37°C in a humid atmosphere with 5% CO_2_. After that, 96-well plates were coated with 100 *μ*L of the capture monoclonal antibody anti-IL-1*β* and incubated for 18 hours at 37°C. The plate was then washed with washer buffer (PBS/Tween20). After that, 200 *μ*L of blocking buffer, containing 5% bovine serum albumin (BSA) in PBS/Tween20, was added to the wells and the plates were incubated for 1 hour at 37°C. Following this, the wells were washed and 50 *μ*L of either samples or standard were dispensed into each well and the plates were incubated for 2 hours at 37°C. After this, the plate was washed and 100 *μ*L of detection antibody anti-IL-1*β* was added for 2 hours at 37°C. After incubation and washing, 100 *μ*L of streptavidin-peroxidase was added, followed by incubation and addition of the substrate (100 *μ*L/mL 3,3′,5,5′-tetramethylbenzidine). Finally, sulfuric acid (50 *μ*L) was added to stop the reaction. Absorbances were recorded at 540 and 450 nm and concentration of IL-1*β* was estimated from standard curves prepared with recombinant cytokine. The results were expressed as pg/mL of IL-1*β*.

#### 2.8.10. Antitumor Activity

Cytotoxic activity of isolated PLA_2_s on human T-cell leukemia (JURKAT) and breast adenocarcinoma (SK-BR-3) lines obtained from the American Culture Collection of Cells (ATCC, American Type Culture Collection, Rockville, MD, USA) were investigated (Figure 6). This activity was assayed by MTT staining as described by Mosmann [25] and adapted by Stábeli et al. [21]. Cells were dispensed in 96-well plates at a density of 5 × 10^5^ cells/mL. After 24 h of incubation, the medium was removed and fresh medium, with or without different concentrations of PLA_2_s (BmatTX-I, BmatTX-II, BmatTX-III, or methotrexate), was added to the wells and incubated for another 24 hours. The evaluation of the cytotoxic activity was measured in a spectrophotometer using an interference filter of 570 nm and expressed as a percentage.

#### 2.8.11. Anti-Leishmania Activity

Promastigote forms of *Leishmania amazonensis *(IFLA/BR/67/PH8) were dispensed in a 96-well plate with 1 × 10^5^ cells/well. Different concentrations (3.12, 6.25, 12.5, 25, 50, and 100 *μ*g/mL) of the crude venom of *B. mattogrossensis* and the isolated PLA_2_s (BmatTX-I and BmatTX-II) were added to each well. 100 mg/mL of pentamidine was used as a positive control. After an incubation period of 48 h, 10 *μ*L of a 5 mg/mL MTT solution was added. Then, the plates were placed in the oven at 33°C with 5% CO_2_ for 4 hours of incubation after which 50 *μ*L of SDS (20%, w/v) was added. Absorbance was monitored at 570 nm. Results were expressed in toxicity percentage following the equation: 1 − (D.O. sample/D.O. control) × 100.

### 2.9. Statistical Analysis

Results were expressed as mean +/− standard deviation. An ANOVA test was used to evaluate the significance of the differences observed with  *P*  value ≤ 0.05 considered to be significant.

## 3. Results and Discussion

### 3.1. Crude Venom Biological Activities

*B. mattogrossensis* snake venom induced hemorrhage, coagulation, proteolytic, and phospholipase activities *in vitro* (Table 1). The hemorrhagic activity of *B. mattogrossensis* crude venom was evaluated based on the dimensions of the average hemorrhagic halo which was 3.33 cm^2^. This result is in agreement with the results recently obtained with *B. atrox* [26].

Coagulation activity was confirmed after incubation of different concentrations of *B. mattogrossensis *crude venom with plasma in which the minimum coagulation dose capable of promoting coagulation in less than 1 min was 0.325 *μ*g of protein. Analysis of the proteolytic activity of *B. mattogrossensis* crude venom demonstrated a concentration-dependent response (data not shown). Regarding phospholipase, proteolytic, coagulating, and hemorrhagic activities of *B. mattogrossensis* crude venom, assays confirmed the presence and activity of proteases and phospholipases. The presence of metalloproteases was evidenced by the important formation of an extensive hemorrhagic halo *in vivo*. The presence of serine proteases was evidenced by coagulating activity present even in low concentrations of the venom (325 *μ*g/mL).

Phospholipase activity of *B. mattogrossensis* crude venom assayed with 4N3OBA synthetic substrate was 1,864.05 U/mg measured by the number of moles of chromophores released per minute (n°mols/min or U) for a given quantity of protein in milligrams (mg).

The properties of snake venom components observed in this study are characteristic of accidents caused by snakes of the *Bothrops sp* genus; symptoms such as pain, edema, hemorrhage, and necrosis and, additionally, systemic disturbances are characteristic and corroborate the literature that describes that proteases of snake venom proteins are closely related to interference in the hemostatic system promoting blood coagulation, fibrinolysis, and platelet aggregation [27–30].

### 3.2. Isolation and Biochemical Characterization of Phospholipases

**Figure 1:**
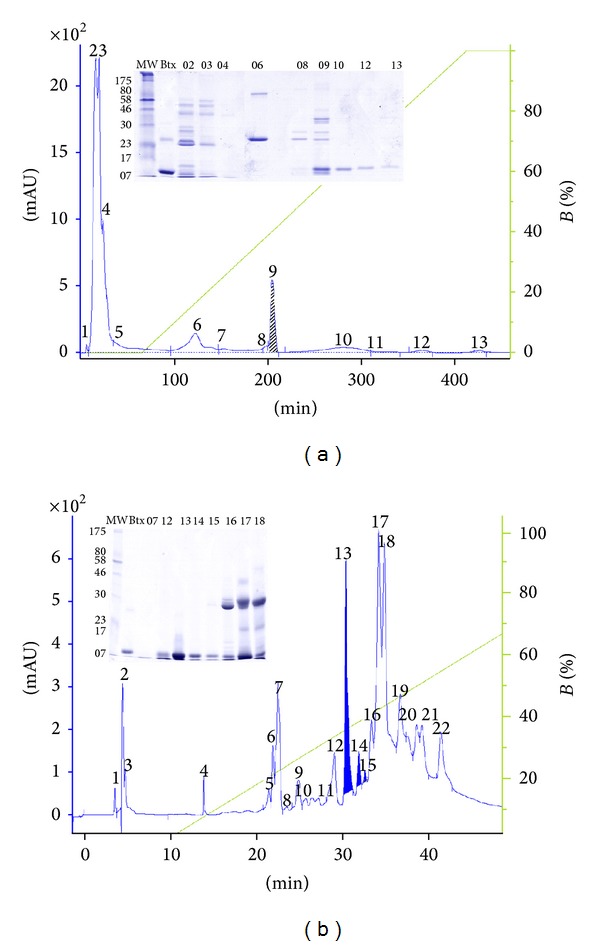
Chromatographic and electrophoretic profile of *Bothrops mattogrossensis* venom fractioning. (a) CM-Sepharose columns equilibrated with solvent A (50 mM ammonium bicarbonate, pH 8.0) and eluted with a 0–100% concentration gradient of solvent B (500 mM ammonium bicarbonate, pH 8.0) at a 5.0 mL/minute flow. Emphasis on peak 9, rechromatographed. (b) Rechromatography of fraction 9 on Discovery C18 column equilibrated with solvent A (0.1% TFA) and eluted with a concentration gradient of 0–100% of solvent B (99.9% acetonitrile and 0.1% trifluoroacetic acid) and a 1.0 mL/min flow. Emphasized in blue are fractions 13, 14, and 15 characterized as phospholipases BmatTX-I, BmatTX-II, and BmatTX-III, respectively. Controls: MW: molecular weight standard; BTx: BthTX-I a basic enzymatically inactive PLA_2_ (Lys49) (10 *μ*g) isolated from *Bothrops jararacussu* venom. Absorbances read at 280 nm. Electrophoresis gel made with 12.5% (w/w) acrylamide/bis-acrylamide.

The present study showed, for the first time, the isolation of the three phospholipases A_2_ from *B. mattogrossensis* snake venom, obtained by two chromatographic steps. First, ionic exchange chromatography was performed on a CM-Sepharose column with an ammonium bicarbonate gradient (50 to 500 mM, pH 8.0). The elution of absorbed proteins with a linear gradient of concentrated buffer resulted in thirteen fractions (Figure 1(a)), of which fraction nine (9) was related to PLA_2_s because it showed phospholipase activity of 221.09 U/mg on artificial substratum. All fractions were lyophilized and submitted to unidimensional electrophoresis revealing many protein bands.

Fraction 9 was submitted to the second chromatographic step in a reverse column phase on a Discovery C18 column, using 0.1% Trifluoroacetic (TFA) and 99.9% Acetonitrile (ACN) as solvents for the separation of other venom components. The elution of absorbed proteins with a linear gradient of concentrated buffer resulted in twenty-two (22) fractions (Figure 1(b)). The PLA_2_s were highly purified with approximately 40% ACN. Similar results were observed in the isolation of other PLA_2_s from snake venoms in high performance liquid chromatography on reverse phase columns where the elution profile of these proteins occurs between 30 and 40% ACN [5].

The association of chromatographic techniques such as ionic exchange and reverse phase has commonly been used, and many snake venoms have been fractioned this way, highlighting the phospholipase purification of a species belonging to the old complex “*Bothrops neuwiedi*”, as well as the target species studied in this research. Two PLA_2_ basic isoforms from *B. (neuwiedi) pauloensis* venom were purified by Rodrigues et al. [31], using biochemical techniques similar to the ones used in this study, with the combination of ionic exchange (cationic) and reverse phase chromatographies.

Other PLA_2_s have also been purified using simplified procedures based in CM-Sepharose and/or reverse phase, as for example, venom PLA_2_s from *Bothrops moojeni* [21, 32, 33], *B. pirajai* [34, 35], *B. jararacussu* [36, 37], *B. alternatus* [8, 38], *Cerastes cerastes* [39], and *Elaphe climacophora *[40].

**Figure 2:**
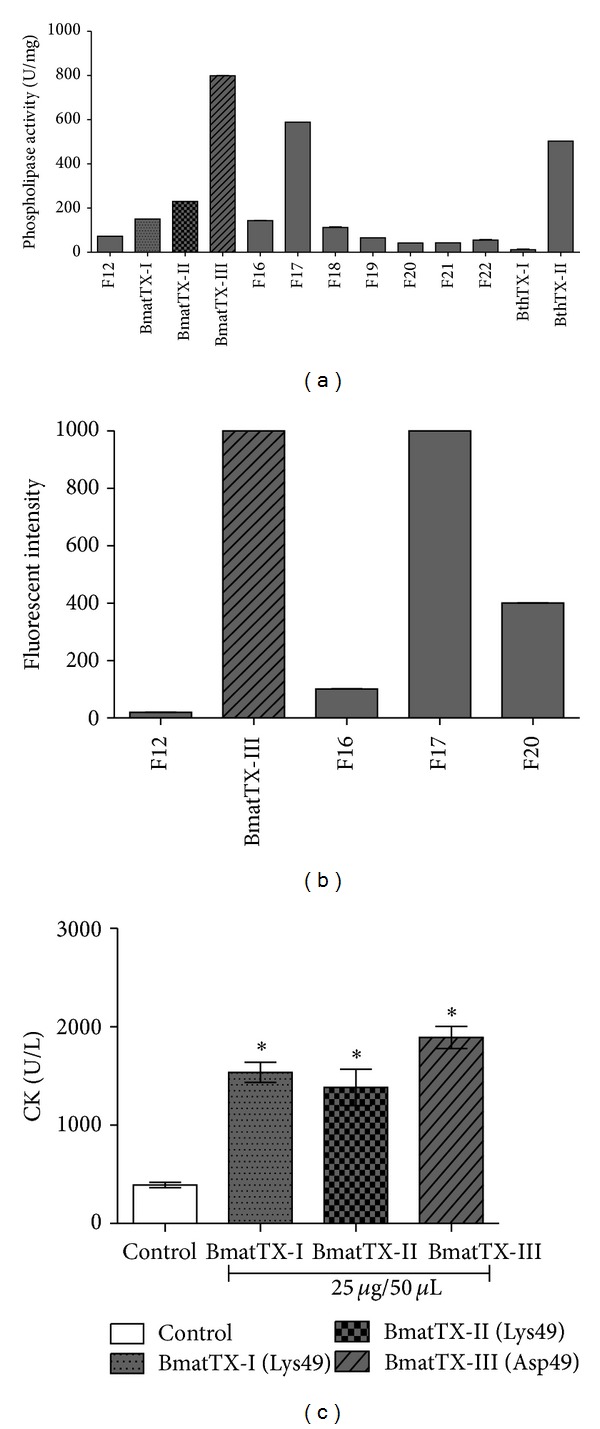
Enzymatic activity and myotoxic activity of PLA_2_s isolated from the venom of *B. mattogrossensis*. (a) Phospholipase activity of the fractions collected from the rechromatography of fraction 9 done in C18 column assayed using an NOB stained substrate. This activity was assessed through the measurement of the number of moles of the released chromophore per minute (n°mols/min or U) per milligram of protein. (b) Phospholipase activity of the fractions collected from the rechromatography of fraction 9 done in C18 column assayed using a fluorescent substrate. (c) Myotoxic activity evaluated for inoculation of PLA_2_s (25 *μ*g/50 µL) or PBS (control) done intramuscularly, in the gastrocnemius muscle of mice. After 3 hours, the creatine kinase (CK) level, an important marker of muscular lesion, was assayed in the animal's plasma. Each bar represents the average +/− SD of three independent groups. *P < 0.05 compared to the control. F13: BmatTX-I (Lys49); F14: BmatTX-II (Lys49); F15: BmatTX-III (Asp49); Controls: BthTX-I (Lys49) and BthTX-II (Asp49).

Fractions 13 and 14 were related to enzymatically inactive PLA_2_s, whereas fraction 15 was related to enzymatically active PLA_2_s. The phospholipase activity of the collected fractions were analyzed with synthetic NOB and NBD-PC substrates (Figures 2(a) and 2(b)). The amount of activity was compared to BthTX-II, a basic enzymatically active PLA_2_ (Asp49), and BthTX-I, a basic enzymatically inactive PLA_2_ (Lys49), both previously isolated from *Bothrops jararacussu* venom [36, 41]. The BmatTX-III PLA_2_s (Asp49) were not able to induce platelet aggregation and did not inhibit collagen or ADP induced platelet aggregation (data not shown).

**Figure 3:**
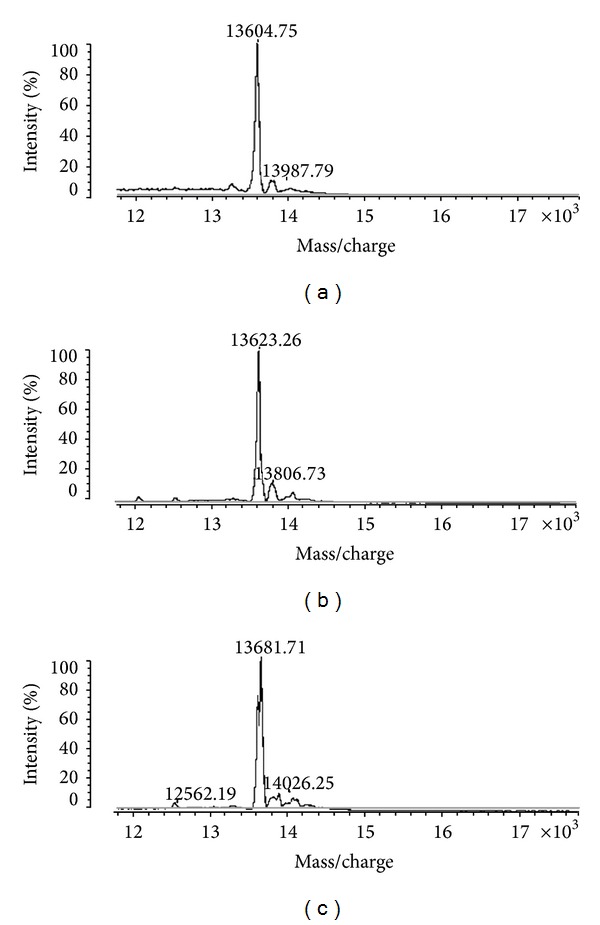
Mass determination by spectrometry. The average protein masses of BmatTX-I (a), BmatTX-II (b), and BmatTX-III (c) were obtained in a MALDI-TOF system, operated in linear mode using external standards for calibration. The resulting mass spectra were submitted to automatic baseline subtraction.

**Figure 4:**
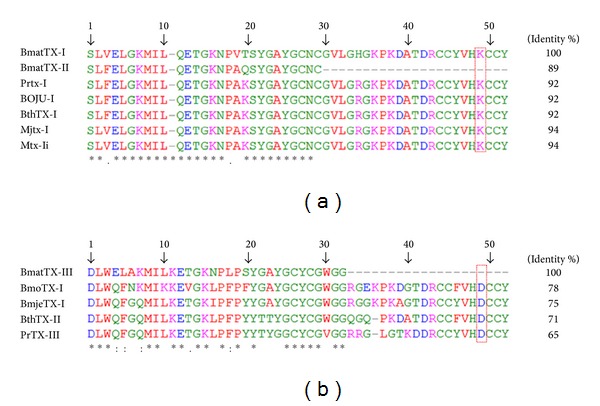
Comparison of the N-terminal sequence of the snake's PLA_2_s with the PLA_2_s isolated from *B. mattogrossensis *venom. N-terminal sequencing of the PLA_2_s obtained by Edman [13] degradation and alignment done with CLUSTALW2 expressed as % of similarity. (a) BmatTX-I, BmatTX-II with Lys49 residues in marked sequences. (b) BmatTX-III with Asp49 residues in marked sequences. Sequences used for the alignment and their respective access numbers: BthTX-I (gi:51890398); MjTX-I (gi:17368325); Mtx-II (gi:390981003); PrTX-I (gi:190016174); BOJU-I (gi:209572966); BthTX-II (gi:1171971); PrTX-III (gi:90016174); BmjeTX-I (gi:313471399); BmoTX-I (gi:221272396). BthTX-I (Lys49), BthTX-II (Asp49), and BOJU-I (Lys49): isolated from the venom of* B. jararacussu*; MjTX-I (Lys49), BmoTX-I (Asp49), and BmjeTX-I (Asp49): isolated from the venom of *B. moojeni*; Mtx-II (Lys49): isolated from the venom of *B. brazili*; PrTX-I (Lys49) and PrTX-III (Asp49): isolated from the venom of *B. pirajai*; the “∗” was used to indicate the amino acid residues that are the same between the sequences.

The degree of purity of the isolated proteins was further demonstrated by SDS-PAGE, mass spectrometry (Figure 3), and N-terminal sequencing (Figures 4(a) and 4(b)). The purified PLA_2_s were named BmatTX-I, BmatTX-II, and BmatTX-III. They were characterized as single polypeptide chains, with isoelectric points around 10 (data not shown). This result agrees with data from published literature, where most basic PLA_2_s, with or without catalytic activity on artificial substrates [32, 42], show an isoelectric point between 8 and 10.

The average molecular mass defined by mass spectrometry was 13,304 Da for BmatTX-I, 13,623 Da for BmatTX-II, and 13,681 Da for BmatTX-III (Figure 3). This molecular mass is consistent with most isolated PLA_2_s from snake venoms which are around 13 to 16 kDa [27, 36, 43].

BmatTX-I sequencing showed a lysine (Lys) at position 49 and although only the first 28 amino acid residues of BmatTX-II have been sequenced, both are highly similar to the PLA_2_ Lys49 homologue subgroup. The N-terminal sequence alignment of BmatTX-I and BmatTX-II has revealed that these basic proteins are PLA_2_s similar to homologous Lys49 and other Lys49 myotoxins from snake venoms (Figure 4(a)). BmatTX-I showed 94% similarity with MjTX-I present in *B. moojeni *venom, 92% with BthTX-I and BOJU-I present in *B. jararacussu* venom, and 94% similarity with MTX-II present in *B. brazili *venom.

Additionally, multiple sequence alignment of BmatTX-III showed a PLA_2_-Asp49 basic myotoxin with another Asp49 of the *Bothrops* genera (Figure 4(b)). It can be observed that BmatTX-III presented 78% similarity with BmjeTX-I and 75% with BmoTX-I, both from *B. moojeni* venom, 71% similarity with BthTX-II isolated from *B. jararacussu* venom, and 65% similarity with PrTX-III from *B. pirajai *venom.

In the analysis of the sequences of the PLA_2_s isolated from *B. mattogrossensis* in this study, highly preserved constituent residues of the *α*-helix structure, characteristic of phospholipases were identified, as well as the presence of many cysteine residues, which suggests the existence of many disulfide bridges, important for the stabilization of PLA_2_'s molecular structure [21, 44].

Some studies regarding the amino acid composition of PLA_2_s demonstrate that these proteins are rich in basic and hydrophobic amino acids containing three long *α*-helixes, two beta sheets, and a Ca^2+^ binding site [45–47]. Calcium is absolutely necessary for hydrolysis; therefore, almost all PLA_2_s have a highly preserved region for a Ca^2+^ bond (XCGXGG) and a catalytic site (DXCCXXHD) [48]. It is observed in BmatTX-III that the calcium binding site (_27_Y**CG**W**GG**_32_) is preserved, indicating that it is a catalytically active PLA_2_ belonging to the PLA_2_ Asp49 subgroup.

### 3.3. Biological Activities of Phospholipases A_2_

The main objective for the isolation and characterization of components of snake venom is to better understand the participation of each component in different pathophysiological processes resulting from envenomation. Local lesions can be attributed to proteases, phospholipase activity, and hemorrhagic factors of these venoms, followed by the release of vasoactive agents causing hemorrhaging in various organs and tissues [5, 44, 49–52].

The PLA_2_s isolated from *B. mattogrossensis* venom, BmatTX-I, BmatTX-II, and BmatTX-III, showed high myotoxic activity (Figure 2(c)). At a concentration of 25 µg/50 µL, the PLA_2_s induced a significant release of CK, an important muscular lesion marker, when compared to the control. Myotoxicity is the characteristic presented by most basic phospholipases A_2_ from snake venoms. Several studies demonstrate that the myotoxic effect begins quickly by direct action of the myotoxic PLA_2_s on the plasma membrane of muscle cells or it is mediated by metalloproteases, due to consequent degeneration and ischemia [27, 28, 44].

Regarding the Lys49-PLA_2_ myotoxins, it is evident that they lyse the plasma membrane of the muscle cell infected *in vivo*; however the exact mechanism has not been described yet. Furthermore, it is not known if the toxin is internalized before, during, or after the initial lysis or if it is not internalized. Although myotoxicity can be induced by the production of fatty acids, there is a second mechanism that seems to be independent of the enzymatic activity and is mediated by the C-terminal region at sites 115–129 of the Lys49 molecules [43, 53, 54].

In an attempt to better understand the development of the inflammatory process unleashed by the protein complex present in snake venoms, many studies have been done, such as edema induction [55], leukocyte participation [56, 57], mast cells degranulation [55], participation of various cytotoxins in the inflammatory system [58, 59], participation of cyclooxygenases [60, 61], and the participation of venom PLA_2_s in the inflammatory process [6, 51, 56, 62].

Many studies about PLA_2_ activity in macrophages have already been done [8, 63, 64]. Little is known about PLA_2_'s effect on neutrophils however. Escocard et al. [65] described an influx of inflammatory cells including many neutrophils into the peritoneal cavity of mice after the injection of* Bothrops atrox *venom. The induction of reactive oxygen species (ROS), cytokines like IL-6 and IL-1*β*, was seen in these neutrophils. These data were also observed in a study carried out by Souza et al. [26] where besides the influx of neutrophils into the peritoneal cavity of mice after injection of the venom of *B. atrox*, there was also induction of superoxide by these cells, mast cell degranulation, and phagocytosis by macrophages. Regarding the activity of PLA_2_ Gambero et al. [66] have observed the ability of some myotoxins (bothropstoxin-I,-II and piratoxin-I) to induce neutrophil chemotaxis in a concentration-dependent manner.

In order to evaluate the activation of leukocytes, the toxicity of *B. mattogrossensis *myotoxins on neutrophil cells was investigated. The cells were incubated with different concentrations (3, 6, and 12 *μ*g/mL) of BmatTX-I (Lys49), BmatTX-II (Lys49), and BmatTX-III (Asp49) myotoxins during 1, 12, and 24 hours (data not shown). These myotoxins did not affect the neutrophils' viability, which agrees with Zuliani et al. [64], showing low toxicity on thioglycollate elicited mice macrophages.

**Figure 5:**
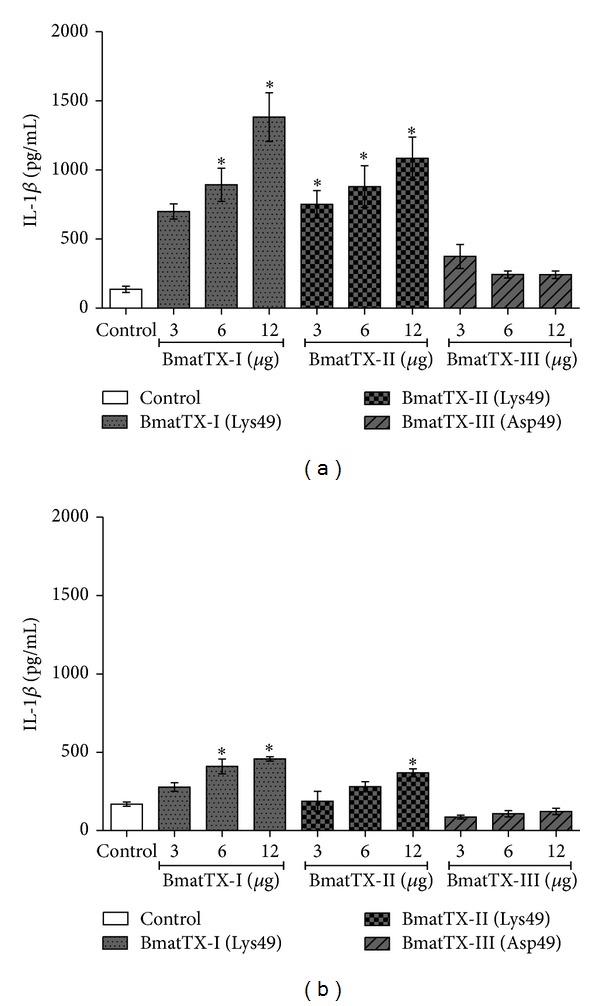
Production of IL-1*β* by mice neutrophils. These cells were incubatedwith PLA_2_ (3, 6, and 12 *μ*g/mL) or RPMI (control) for 12 hours (a) and 24 hours (b) at 37°C in a humid atmosphere with 5% CO_2_. Quantification was done by ELISA as described in 2.9. Each bar represents the average +/− SD of three independent groups. *P < 0.05 compared to the control.

**Figure 6:**
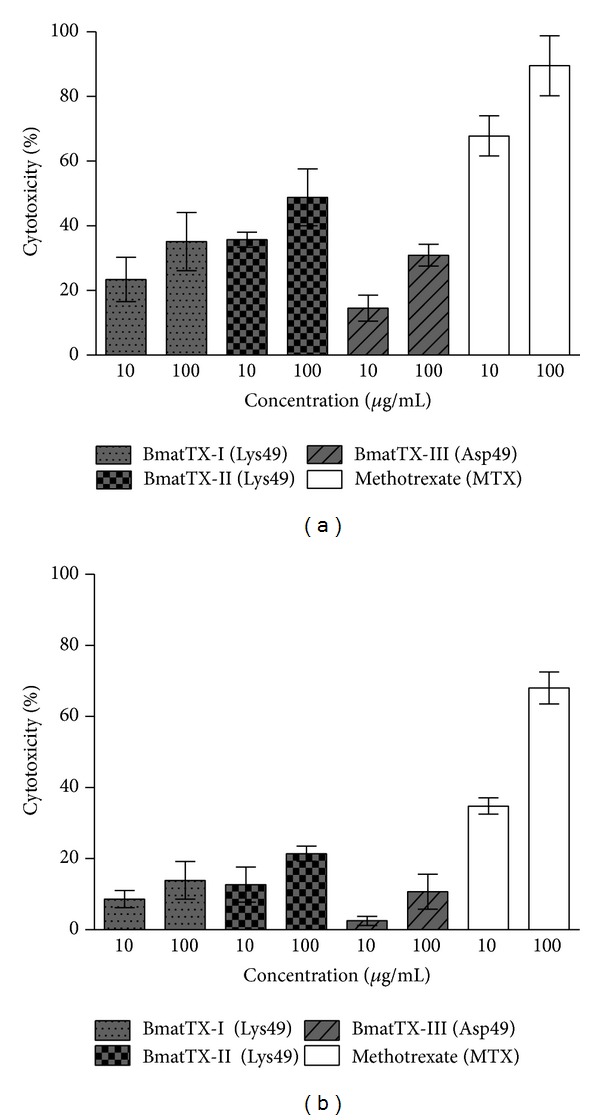
Antitumoral activity of PLA_2_s from *B. mattogrossensis*. (a) Antitumoral activity on human acute T-cell leukemia (JURKAT) lines. (b) Antitumoral activity on human breast adenocarcinoma (SK-BR-3). Different concentrations of the PLA_2_s were incubated with cell lines. Methotrexate was used as the positive control. Results are presented as mean +/− SD (*n* = 3).

Nonetheless, our data showed that neutrophils incubated with BmatTX-I and BmatTX-II myotoxins induced the release of IL-1*β* (Figures 5(a) and 5(b)). Moreover, these results suggest that phospholipid hydrolysis is not essential for the activity observed and argue with the hyphotesis that other molecular regions distinct from the active site may be involved in this effect.

PLA_2_s are multifunctional proteins that can be used as mediators in several areas of medicine, such as in the treatment of rheumatoid arthritis, as a new class of HIV inhibitors by blocking the host cell invasion, as a potential treatment against malaria, and as an antibiotic by inducing cytotoxicity via the disruption of bacterial membranes [21, 23, 30, 67, 68]. A study by Costa et al. [67] with PLA_2_s isolated from *B. brazili*, MTX-I and II, demonstrated cytotoxic activity against Jurkat tumor cells as well as antimicrobial activity against *E. coli* and *C. albicans* and antiparasitic activity against *Leishmania sp*.

In the present study we evaluated the cytotoxic activity of PLA_2_s isolated from *B. mattogrossensis *on JURKAT (T leukemia) and SK-BR-3 (breast adenocarcinoma), both of which are human tumor cell lines. Like MTX-II of *B. brazili* [67] the cytotoxic activity of PLA_2_s BmatTX-I and BmatTX-II on JURKAT cells was independent of their catalytic activity, since these are characterized as Lys49 PLA_2_s and are therefore catalytically inactive. BmatTX-III, characterized as Asp49, despite being enzymatically active also showed a lower level of toxicity. Some authors propose that the cytotoxic activity on tumor cell lines is associated with the induction of apoptosis, considering the fact that PLA_2_ promotes alterations in the cell membrane. And some studies involving Lys49 PLA_2_s isolated from *B. asper *demonstrated that the C-terminal region comprised of amino acids 115–129 is concerned with the cytotoxic and bactericidal activities of this protein [69–71]. The same observation was made by Costa et al. [67] with synthetic peptides derived from the C-terminal portion of MTX-I and II PLA_2_s.

**Figure 7:**
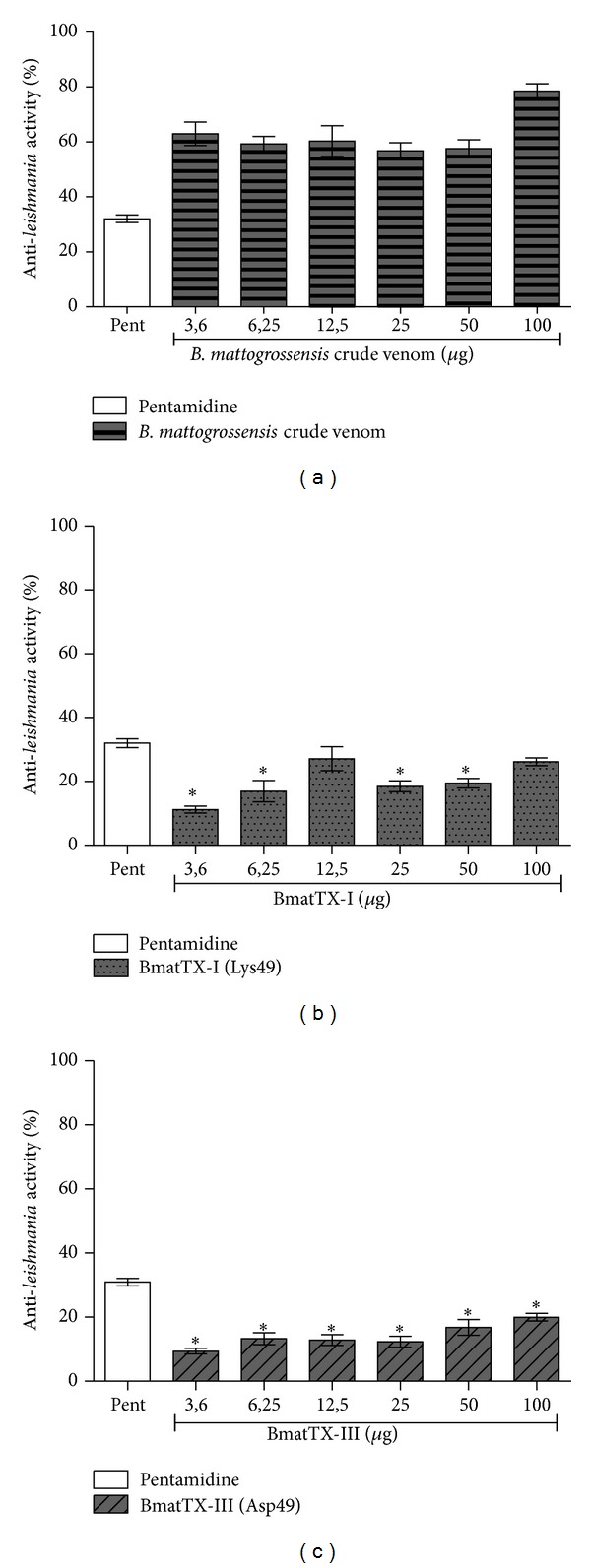
Antileishmanial activity of the crude venom of and PLA_2_s from *B. mattogrossensis*. The promastigote forms of *L. amazonensis* were plated with 1 × 10^5^ cells/well. Then, different concentrations of crude venom and isolated PLA_2_s were added to each well. The experiment was done in a 48 h period, with the (a) crude venom and the PLA_2 _enzymes, (b) BmatTX-I, and (c) BmatTX-III. MTT was added and after the incubation period at 33°C, the formazan crystal formed was dissolved in SDS. Readings were done in a spectrophotometer at 570 nm. Each bar represents the average +/− SD of the three independent experimental groups, sixfold total. *P < 0.05 compared to the control.

We also evaluated the antiparasitic activity of the crude venom and isolated PLA_2 _of *B. mattogrossensis* on promastigote forms of *L. amazonensis*. After the analysis, it was observed that the crude venom of *B. mattogrossensis* presents increasing toxic activity (approximately 50 to 80%) against promastigote forms of *L. amazonensis* after 48 h of incubation (Figure 7(a)). The PLA_2_s isolated from *B. mattogrossensis, *at 100 *μ*g/mL, characterized as BmatTX-I (Lys49) and BmatTX-III (Asp49) presented toxic activity between 25% and 30%, respectively, even with values close to those presented after incubation of the protozoan with Pentamidine, a drug used as a positive control (Figures 7(b) and 7(c)).

When compared, the cytotoxicity values of PLA_2_s against promastigote forms of *L. amazonensis* show similar activity between the Lys49-PLA_2_ and the Asp49-PLA_2_. Comparison of this activity with the crude venom (70%) showed that PLA_2_s are responsible for almost half of the observed effect. Nonetheless, notably, the results suggest that other toxins present in the venom contribute to the parasite's death.

Similar to the results obtained in the present study, Stábeli et al. [21] demonstrated that MjTX-II, a Lys49-PLA_2_ homologue isolated from *B. moojeni* venom, in different concentrations (5, 25 and 75 *μ*g) was effective as a parasiticide agent against *Schistosoma mansoni* and promastigote forms of *Leishmania* (*L. amazonensis, L. braziliensis, L. donovani, *and* L. major*).

The action of the PLA_2_s, BmatTX-I (Lys49), and BmatTX-III (Asp49), on promastigote forms of *L. amazonensis,* was independent of its catalytic activity, since catalytically inactive Lys49 myotoxin also demonstrated toxicity against *Leishmania*. It is believed that the observed cytotoxic activity might be related to the C-terminal regions of these phospholipase-homologues that are able to promote a disturbance in the cellular membranes independent of their catalytic activity [67, 72]. However, more studies are necessary to define the exact mechanism of action of these enzymes on parasites.

Growing interest in the comprehension of the structure and function of snake venom components, especially PLA_2_, contributes to a better understanding of the mechanism of action of their enzymatic and toxic activities. It opens the path to better understand the intoxication caused by envenomation and the physiopathology behind its side effects. Future studies will potentially improve serum therapy and help develop the pharmaceutical potential that molecules isolated from animal venoms can have, such as the PLA_2_s isolated from the *B. mattogrossensis* venom which show anti-*Leishmania *and antitumor activities.

## 4. Conclusion

In conclusion, the venom of *Bothrops mattogrossensis* has a qualitatively similar toxicological profile to previously studied snake venoms of the *Bothrops sp.* genera despite the observation of quantitative variations. Of the three basic PLA_2_s from *B. mattogrossensis *venom, now isolated for the first time, two are characterized as Lys49-PLA_2_ homologues, BmatTX-I and -II, and the other as an Asp49-PLA_2_, named BmatTX-III. This showed high phospholipase activity. The PLA_2_s isolated induced myotoxic effects as well as the release of proinflammatory cytokines by neutrophils. BmatTX-I and -III PLA_2_s were cytotoxic to human tumor cell lines JURKAT and SK-BR-3 and showed activity against promastigote forms of* L. amazonensis.*

## Figures and Tables

**Table 1:** Activities induced by *B. mattogrossensis* snake venom.

Effect^a^	Activity
Phospholipase activity^b^ (U/mg)	1,864.05
Proteolytic activity^c^ (U/10 *μ*g)	3.0 ± 0.1
Hemorrhagic halo^d^ (cm^2^)	3.33 ± 0.05
Coagulation activity^e^ (MCD, *μ*g)	0.325

^a^All experiments were carried out in triplicate. ^b^Activity using NOB stained substrate. ^c^One enzymatic unit (U) was defined as the quantity of enzyme needed to increase the absorbance by 0.05 UA/440 nm. ^d^Values 3 h after incubation with crude venom of *B. mattogrossensis* (50 *μ*g). ^e^MCD: the minimum coagulation dose was the dose capable of coagulating 200 *μ*L of citrated plasma in less than a minute.
